# Síndrome Pós-COVID ou COVID Longa: Um Novo Desafio para o Sistema de Saúde

**DOI:** 10.36660/abc.20230750

**Published:** 2023-12-14

**Authors:** José Geraldo Mill, Jéssica Polese

**Affiliations:** 1 Departamento de Ciências Fisiológicas Centro de Ciências da Saúde Universidade Federal do Espírito Santo Vitória ES Brasil Departamento de Ciências Fisiológicas - Centro de Ciências da Saúde - Universidade Federal do Espírito Santo , Vitória , ES – Brasil; 2 Programa de Pós-Graduação em Ciências Fisiológicas Universidade Federal do Espírito Santo Vitória ES Brasil Programa de Pós-Graduação em Ciências Fisiológicas - Universidade Federal do Espírito Santo , Vitória , ES – Brasil; 3 Faculdade Brasileira Univix Vitória ES Brasil Faculdade Brasileira Univix , Vitória , ES – Brasil

**Keywords:** COVID-19, Síndrome Pós-COVID-19 Aguda, Sistemas de Saúde

A COVID-19 representou um desafio sem precedentes para a saúde pública em todo o mundo. Segundo o *‘WHO Coronavirus (Covid-19) Dashboard* ’ (atualizado em 18/10/23), a doença afetou 770 milhões de pessoas e causou 6,9 milhões de óbitos. ^1^ Em número de casos o Brasil ocupa a 8 ^a^ posição (38 milhões), subindo para a 2 ^a^ em número de óbitos (mais de 700 mil) e, infelizmente, alcança o 1 ^o^ na taxa de letalidade com, aproximadamente, 368 óbitos/100 mil habitantes. Esses dados indicam que, se a pandemia foi devastadora para o mundo, foi ainda mais drástica para a população brasileira. A letalidade da doença a nível mundial foi de cerca de 1% e, também neste indicador, o Brasil tem destaque negativo com letalidade próxima a 1,9%. Como a doença afetou um número enorme de indivíduos, também é grande o contingente sobrevivente à doença.

A maior parte dos indivíduos afetados pelo SARS-CoV-2 se recupera plenamente após a doença. Uma parcela importante, entretanto, permanece sintomática por um período variável de tempo. ^2^ A persistência de sintomas é mais frequente e variada nas formas mais graves da doença. Numa coorte de 41 pacientes que necessitaram internação hospitalar por, em média, 9 dias, 80% apresentavam função pulmonar diminuída 30 dias após alta, a qual persistiu por até 6 meses em cerca de 10% dos pacientes, alguns deles com sinais claros de síndrome pulmonar restritiva, fator limitante para o esforço físico. ^3 , 4^

A persistência de sintomas levou a Organização Mundial da Saúde a definir a *síndrome pós-Covid* ou *Covid longa* como uma nova entidade clínica que aparece em pacientes recuperados da infecção pelo SARS-CoV-2. A síndrome se caracteriza pela persistência, por mais de 3 meses, de sintomas que não podem ser explicados por uma condição prévia à infecção viral. ^5^ Adotando-se essa definição, vários estudos mostram que até 30% dos casos desenvolvem a síndrome. ^2^ A dificuldade de caracterizar a síndrome decorre da grande variedade de sintomas. A subjetividade destes sintomas, entretanto, dificulta a exclusão da sua existência antes da doença. Em metanálise de 76 estudos, os sintomas mais frequentemente reportados foram fadiga (37,8%), desconforto pós-esforço (35,5%), distúrbios do sono (25,2%), dispneia (23,4%), ansiedade (21,7%), confusão mental (13,4%), depressão (13,1%), dificuldade de se concentrar (13,1%) e alterações do paladar (11,2%). ^6^ Mas uma miríade de outros sintomas também foram relatados. Essa variedade dificulta a identificação de possíveis mecanismos. Várias hipóteses têm sido levantadas, incluindo desregulação do sistema imune, persistência prolongada do vírus, instalação ou agravamento de estado inflamatório crônico ou ainda que os sintomas seriam secundários a lesões em órgãos específicos afetados pelo vírus, incluindo sistema nervoso, coração/vasos sanguíneos, rins, etc. ^7^ Alguns autores têm tentado agregar os sintomas (clusters) e três agregados têm sido propostos como os mais comuns. ^6^ O mais comum seria vinculado a alterações cardiovasculares decorrentes de sequelas cardíacas ou vasculares pós-infecção que levam aos sintomas que predominam na listagem acima, como desconforto pós-esforço, fadiga muscular e dispneia. Outro cluster seria constituído por alterações no sistema nervoso, onde predominam os distúrbios do sono, a ansiedade e a depressão. Um terceiro teria sintomas mais difusos secundários à persistência, a longo prazo, do estado inflamatório da fase aguda.

Os sintomas variam em função de comorbidades pré-existentes, da idade e da gravidade da doença. Um estudo multicêntrico encontra-se em andamento no Brasil visando quantificar o impacto da COVID-19 leve ou moderada na qualidade de vida e na função cardiovascular de pacientes. ^8^ Neste número dos Arquivos Brasileiros de Cardiologia, Trott et al. apresentam um artigo metodológico de estudo multicêntrico que concentrará esforços para elucidar o quadro sintomatológico ao longo do tempo em pacientes acometidos de COVID-19 moderada a grave visando correlacionar os sintomas ao longo de um ano com o quadro clínico da fase aguda da doença. Serão incluídos apenas pacientes que tiveram assistência hospitalar especializada na fase aguda da doença. ^9^ Apesar de a coleta de dados não ser presencial, o que seria ideal, a seleção rigorosa de pacientes e o acompanhamento aos 3, 6, 9 e 12 meses após a alta constitui esforço relevante para obter melhor entendimento da dimensão do problema visando propor mecanismos que possam explicar a diversidade de sintomas e abrir pistas para tratamentos mais específicos. O Estudo Longitudinal de Saúde do Adulto (ELSA-Brasil) tem obtido alguns dados importantes nesse sentido, notadamente em função de variáveis de saúde mental, uma vez que o estudo dispõe de dados dos mesmos indivíduos e com os mesmos métodos antes e após a pandemia. ^10^ Análises de dados dos participantes do ELSA-Brasil que foram acometidos ou não pela Covid-19 podem fornecer dados importantes para uma melhor compreensão do impacto da doença em vários órgãos, sistemas e comportamentos. A COVID-19 representou um enorme desafio para os sistemas de saúde em todo mundo. Desafio não menos importante deverá ser enfrentado na abordagem das sequelas físicas e psíquicas deixadas pela pandemia.
