# Supplementary material for: Síndrome Pós-COVID ou COVID Longa: Um Novo Desafio para o Sistema de Saúde
Source: Arq Bras Cardiol. 2023 Dec 14;120(11):e20230750. [Article in Portuguese] doi: 10.36660/abc.20230750 (PMC10763636; doi:10.36660/abc.20230750)
Supplement: Translation

# Translation: Post-COVID Syndrome or Long COVID: A New Challenge for the Healthcare System

**Authors:** José Geraldo Mill, Jéssica Polese

**Affiliations:** 1 Brazil Departamento de Ciências Fisiológicas - Centro de Ciências da Saúde - Universidade Federal do Espírito Santo, Vitória, ES – Brazil; 2 Brazil Programa de Pós-Graduação em Ciências Fisiológicas - Universidade Federal do Espírito Santo, Vitória, ES – Brazil; 3 Brazil Faculdade Brasileira Univix, Vitória, ES – Brazil

**Keywords:** COVID-19, Post-Acute COVID-19 Syndrome, Health Systems

COVID-19 posed an unprecedented challenge to public health around the world. According to the ‘WHO Coronavirus (Covid-19) Dashboard’ (updated on 10/18/23), the disease affected 770 million people and caused 6.9 million deaths. ^1^ In terms of the number of cases, Brazil occupies the 8 ^th^ position (38 million), rising to 2 ^nd^ in the number of deaths (more than 700 thousand) and, unfortunately, reaches 1 ^st^ in fatality rate with approximately 368 deaths/100 thousand inhabitants. These data indicate that if the pandemic was devastating for the world, it was even more drastic for the Brazilian population. The lethality of the disease worldwide was around 1%, and, also in this indicator, Brazil stands out negatively, with lethality close to 1.9%. As the disease affected a huge number of individuals, the number of survivors of the disease is also large.

Most individuals affected by SARS-CoV-2 fully recover after illness. An important portion, however, remains symptomatic for a variable period of time. ^2^ The persistence of symptoms is more frequent and varied in more severe forms of the disease. In a cohort of 41 patients who required hospitalization for, on average, 9 days, 80% had reduced lung function 30 days after discharge, which persisted for up to 6 months in around 10% of patients, some of them with clear signs of restrictive pulmonary syndrome, limiting factor for physical effort. ^3 , 4^

The persistence of symptoms led the World Health Organization to define the *post-Covid syndrome* or *Long Covid* as a new clinical entity appearing in patients recovered from SARS-CoV-2 infection. The syndrome is characterized by the persistence, for more than 3 months, of symptoms that cannot be explained by a condition prior to the viral infection. ^5^ Adopting this definition, several studies show that up to 30% of cases develop the syndrome. ^2^ The difficulty in characterizing the syndrome arises from the wide variety of symptoms. The subjectivity of these symptoms, however, makes it difficult to exclude their existence before the disease. In a meta-analysis of 76 studies, the most frequently reported symptoms were fatigue (37.8%), post-exertional discomfort (35.5%), sleep disturbances (25.2%), dyspnea (23.4%), anxiety (21.7%), mental confusion (13.4%), depression (13.1%), difficulty concentrating (13.1%) and changes in taste (11.2%). ^6^ However, a myriad of other symptoms have also been reported. This variety makes it difficult to identify possible mechanisms. Several hypotheses have been raised, including dysregulation of the immune system, prolonged persistence of the virus, installation or worsening of a chronic inflammatory state, or even that the symptoms would be secondary to lesions in specific organs affected by the virus, including the nervous system, heart/blood vessels, kidneys. ^7^ Some authors have tried to aggregate symptoms (clusters), and three clusters have been proposed as the most common. ^6^ The most common would be linked to cardiovascular changes resulting from post-infection cardiac or vascular sequelae that lead to the symptoms that predominate in the list above, such as post-exertion discomfort, muscle fatigue, and dyspnea. Another cluster would be made up of changes in the nervous system, where sleep disorders, anxiety, and depression predominate. A third would have more diffuse symptoms secondary to the long-term persistence of the acute phase inflammatory state.

Symptoms vary depending on pre-existing comorbidities, age, and severity of the disease. A multicenter study is underway in Brazil to quantify the impact of mild or moderate COVID-19 on the quality of life and cardiovascular function of patients. ^8^ In this issue of *Arquivos Brasileiros de Cardiologia* , Trott et al. present a methodological article on a multicenter study that will concentrate efforts to elucidate the symptomatological picture over time in patients suffering from moderate to severe COVID-19, aiming to correlate the symptoms over a year with the clinical picture of the acute phase of the disease. Only patients who received specialized hospital care in the acute of the disease will be included. ^9^ Although data collection is not carried out in person, which would be ideal, the rigorous selection of patients and follow-up at 3, 6, 9, and 12 months after discharge constitute a relevant effort to obtain a better understanding of the dimension of the problem, aiming to propose mechanisms that may explain the diversity of symptoms and open clues to more specific treatments. The Longitudinal Study of Adult Health ( *Estudo Longitudinal de Saúde do Adulto ELSA-Brasil* ) has obtained some important data in this regard, notably in terms of mental health variables, since the study has data from the same individuals and with the same methods before and after the pandemic. ^10^ Analysis of data from ELSA-Brasil participants who were or were not affected by Covid-19 can provide important data for a better understanding of the impact of the disease on various organs, systems, and behaviors. COVID-19 has represented a huge challenge for healthcare systems around the world. No less important challenge must be faced in addressing the physical and psychological consequences left by the pandemic.
